# Nutritional and Functional Assessment of Hospitalized Elderly: Impact of Sociodemographic Variables

**DOI:** 10.1155/2013/101725

**Published:** 2013-10-10

**Authors:** Emam M. M. Esmayel, Mohsen M. Eldarawy, Mohamed M. M. Hassan, Hassan Mahmoud Hassanin, Walid M. Reda Ashour, Wael Mahmoud

**Affiliations:** ^1^Department of Internal Medicine, Faculty of Medicine, Zagazig University, Zagazig 44519, Egypt; ^2^Department of Neurology, Faculty of Medicine, Zagazig University, Zagazig 44519, Egypt

## Abstract

*Background.* This work was constructed in order to assess the nutritional and functional status in hospitalized elderly and to study the associations between them and sociodemographic variables. *Methods.* 200 elderly patients (>65 years old) admitted to Internal Medicine and Neurology Departments in nonemergency conditions were included. Comprehensive geriatric assessments, including nutritional and functional assessments, were done according to nutritional checklist and Barthel index, respectively. Information was gathered from the patients, from the ward nurse responsible for the patient, and from family members who were reviewed. *Results.* According to the nutritional checklist, 56% of participants were at high risk, 18% were at moderate risk of malnutrition, and 26% had good nutrition. There was a high nutritional risk in patients with low income and good nutrition in patients with moderate income. Also, there was a high nutritional risk in rural residents (61.9%) in comparison with urban residents (25%). Barthel index score was significantly lower in those at high risk of malnutrition compared to those at moderate risk and those with good nutrition. *Conclusions.* Hospitalized elderly are exposed to malnutrition, and malnourished hospitalized patients are candidates for functional impairment. Significant associations are noticed between both nutritional and functional status and specific sociodemographic variables.

## 1. Introduction

In recent years, there has been a sharp increase in the number of older persons worldwide [1]. It is estimated that almost half of the adults who are hospitalized are 65 years of age or older, although those older than 65 years represent only 12.5 percent of the population [2]. Aging is associated with various physiological changes and needs, which make elderly people vulnerable to malnutrition [3].

Malnutrition is a major geriatric problem associated with poor health status and high mortality, and the impact of a patient's nutritional condition on the clinical outcome has been widely recognized [4]. Application of nutritional support based on nutritional screening results significantly reduced the incidence of complications and the length of hospital stay [5]. The prevalence of malnutrition varies considerably depending on the population studied and the criteria used for the diagnosis [3].

The nutritional screening checklist (NCL) is the most frequently used nutritional screening tool for community-dwelling older adults. It is intended to prevent impairment by identifying and treating nutritional problems before they become a detriment to the lives of older adults [6]. Nutrition and physical activity impact functional changes through changes in body composition [7].

Tanimoto et al. recently reported that sarcopenia, defined by muscle mass, muscle strength, and physical performance, was associated with functional decline over a 2-year period in elderly Japanese [8]. Also, Andre et al. found that the percentage of elderly with dependence was significantly higher in the malnourished (87.6%) than in well-nourished elderly (50.9%) [3].

Dependency interferes with the health and quality of life, not only for the elderly, but also for relatives and healthcare providers [9]. Assessment of functional capacity is a key element in geriatric health, as it can help in identifying what services or programs are needed [10]. The Barthel self-care index (BSI) is proposed as the standard index for clinical and research purposes [11], and it has proved to be a good predictor of in-hospital mortality [12]. Matzen et al. [13] recently concluded that it is a strong independent predictor of survival in older patients admitted to an acute geriatric unit, and they also suggested that it may have a potential role in decision making for the clinical management of frail geriatric inpatients. It is also found useful for evaluating the functional ability of elderly patients being treated in outpatient care [14].

However, less attention is given to the underlying risk of functional decline and the vulnerability to hospital-associated complications [2]. Also, information about the nutritional and functional status and its correlation with sociodemographic variables in Egypt among hospitalized elderly are scarce; the sociodemographic data will focus a bit on which group is at increased risk of nutritional and functional instability, and hence which group deserves more attention. This study was conducted in order to clarify the impact of sociodemographic factors on nutritional and functional status of the elderly in, addition to the interrelationship between both.

## 2. Methods

This study has been carried out on 200 elderly patients (>65 years old) admitted to Internal Medicine and Neurology Departments, Zagazig University Hospitals, which are educational hospitals serving Sharkia governorate, during the period between October 2009 and October 2010. The admission was due to gastrointestinal troubles in 88 patients, cardiac troubles in 28, chest troubles in 40, neurological in 28, or other causes in 16 patients.

According to age, they were classified into young old (65–74.9 years), old old (75–84.9 years), and oldest old (≥85 years). Patients who required treatment in specialized units such as the intensive care units, were excluded. All participants gave informed consents and the study had approval from local ethical committee.

Information was gathered on admission by the treating physician from the patients, from the ward nurse responsible for the patient, and from family members who reviewed. All the patients were subjected to full history taking and clinical examination.

 Nutritional assessment was done by physical examination by searching for clinical signs of nutritional deficiencies. We used the NCL for nutritional screening which included 10 items with a total score of 21 points. A score from 0 to 2 was considered as good nutrition, 3 to 5 as moderate nutritional risk, and 6 or more as high nutritional risk [15]. The nutritional checklist includes illness and tooth or mouth problems affecting feeding, number of meals per day, and types of foods and drinks. Also ability to eating alone, to cook, shop or feed oneself, and weight gain or loss, are included. One study using the NCL was carried out and the researchers found that, when compared to nutritional assessment criteria (anthropometric, dietary, and laboratory data), the NCL identified 83% of the population as being at high risk compared to 74% using the nutritional assessment criteria [16]. It has been suggested that the NCL is more appropriately used in observational epidemiological studies than for population screening [17].

 Functional assessment was done using the Barthel self-care index of activities of daily living [18]. The index included bowels, bladder, grooming, toilet use, feeding, transfer, mobility, dressing, stairs, and bathing. Total possible scores range from 0 to 20, with lower scores indicating increased disability. Sociodemographic information and nutritional checklist were collected by direct interviews, while Barthel index was collected using chart reading.

### 2.1. Statistical Analysis

Data were checked, entered, and analyzed using SPSS version 15 for data processing and statistics. Continuous variables were tested for normal distribution and expressed as mean ± standard deviation and compared by Student's *t* test. Categorical variables were compared with the chi-square test. For all analyses, *P* value <0.05 was considered statistically significant.

## 3. Results

 Demographic data of the studied groups are presented in Table 1. A total of 200 elderly patients were included in the study. The large majority of patients were illiterate (84%) and none of them were on regular exercise. As for marital state, 56% were widowed and 40% married with only 4% having no children. 56% had low income with only 2% having means of transportation (Table 2).

According to NCL, 56% of the participants were at high risk and 26% had good nutrition (Table 3). Nutritional checklist score was worst in age group ≥85 years, while age has no significant effect on BSI (Table 4). There was a high nutritional risk in rural residents (61.9%) in comparison with urban residents (25%); the mean of BSI was higher in patients from rural than that of urban areas with no significant difference (Table 5).

High nutritional risk was found in 52.4% of illiterate patients and 75% of educated patients, while there was no association between BSI and education (*P* = 0.56) (Table 6). No significant association was found between BSI and gender (*P* = 0.22) and also between NCL and gender (*P* = 0.16) (Table 7).

There was a significant association between NCL and income (*P* = 0.017), as there was a high nutritional risk in patients with low income and good nutrition in patients with moderate income, in contrast there was no association between BSI and income (*P* = 0.47) (Table 8). A significant association between NCL and BSI (<0.001) indicates that both nutrition and functional ability go together (Table 9).

## 4. Discussion

 Older patients tend to have multiple organic, psychological, and social problems [19]. Their functional and physiological capacities are often diminished and the adverse effects of drugs are more pronounced. The concept of Comprehensive Geriatric Assessment (CGA) has evolved because of the many problems of elderly subjects [20]. Our study was concerned with patients admitted to Internal Medicine department with age above 65 years. Majority of age group was young old and represented 88% while the oldest old represented 4% of patients included in the study.

As regards assessment of nutrition, considerable number of studies have examined the nutritional status of institutionalized elderly people and reported prevalence figures for malnutrition and nutritional problems [21]. According to the American Dietetic Association (ADA), the nutrient requirements of elderly peopleare not fully understood, although it is known that the physiological and functional changes that occur with agingcan result in changes in nutrient needs [22].

 The American Academy of Family Physicians and the National Council of Aging in the United States previously formed the Nutrition Screening Initiative for the development of strategies to detect nutritional risks among older people. One of the strategies included the development of a ten-question checklist, the NCL [23]. This checklist, which was designed to be self-administered, could also be administered by a healthcare professional.

 According to NCL, our results showed that about 56% of patients had high nutritional risk. A study conducted in Canada indicated that 37–62% of elderly were at risk of the poor nutrition [24], and the percent was 50.3% in another study in India [25]. A recent study in China [4] found that the overall prevalence of under nutrition and nutritional risk was 17.8% and 41.5%, respectively, using the NCL. Another recent study in Makkah found that among 102 recently hospitalized elderly and according to the mini nutritional assessment (MNA) tool, 22.6% were classified as malnourished, 57.8% were at risk of malnutrition, and 19.6% were well nourished [1]. Also, another study found that 10.2% of elderly individuals were malnourished and 39.9% were at risk of malnutrition according to the MNA screening tool [5]. Differences in prevalence rates of malnutrition among the different studies may be due to difference in selection criteria of elderly, different assessment tools, and differences in sociodemographic variables. 

 In this study, there was an association between NCL and age group. We found that 100% of oldest old had high nutritional risk and 85.7% of old old have high nutritional risk, but the percent in young old was 48.8%. Our results agree with that obtained by Fang et al. [4] who found that the prevalence of nutritional risk was significantly higher in patients >70 years of age than in patients <70 years (64% versus 32%, *P* < 0.001). Yap et al. found that high nutritional risk was more in older patients but with no significant association with age [26].

 Also in this study, we found that high nutritional risk represented 52.4% in illiterate patients and about 75% in educated patients; however, we cannot depend on this finding due to the large difference in sample size between illiterate and educated patients (84% versus 16%, resp.). However, the previous study [26] found no significant correlation between NCL and education, and the percent of patients with high nutritional risk was 26.2% and 33.6% in educated versus illiterate patients, respectively. Increased nutritional risk in males was 64.3% versus 45.5% in females (*P* = 0.16), and this was confirmed by MacLellan and van Til [24]. Contrarily, Rambousková et al. [27] concluded that institutionalized women should be considered a nutritionally vulnerable population group; the reason for this difference may be the higher average age of women versus men (86.1 ± 6.15 versus 81.5 ± 7.97 years) in their study. Meanwhile, Fang et al. [4] found no gender difference in the prevalence of nutritional risk. We found a high nutritional risk in rural residents (61.9%) in comparison with urban residents (25%); this may be due to a higher income and a better nutrition knowledge in urban areas.

High nutritional risk in patients with low income was 60.7%, while it was about 50% in patients with moderate income. A study in Nigeria concluded that males had significantly higher income, higher socioeconomic status scores and were also less vulnerable to malnutrition than females (*P* < 0.05) [28]. Poverty is a nonphysiological cause of under nutrition in older people [29].

 As regards functional assessment, the Barthel self care index of activities of daily living, first developed in 1965 [30] and later modified by Granger et al. [31], measures functional disability by quantifying patient performance in 10 activities of daily life. The Barthel Index has been reported to have excellent reliability and validity and adequate responsiveness to change, in measuring neurologic physical disability. Hobart et al. compared psychometric properties of the Barthel index with newer and lengthier scales, the Functional Independence Measure (FIM) and the Functional Independence Measure + Functional Assessment Measure (FIM+FAM), in patients undergoing rehabilitation. This study suggested that the newer and more extensive rating scales of FIM and FIM+FAM offered few advantages over the more practical and economical Barthel index [32]. Similar results were observed in studies of patients with multiple sclerosis and stroke [33, 34].

In this study, we found a significant correlation between disability and age, where functional activity decreased with advancing age. Hairi et al. showed that advancing age was significantly associated with functional limitation [35]. We did not find significant correlation between disability and income, however, a study done ona random sample of persons, 55–85 years of age, drawn from the population registers of 11 municipalities found that low socioeconomic status had been associated with physical disability [36]. Unless low socioeconomic status is associated with handwork, the elderly will be more susceptible to physical inactivity and lack of exercise.

 Gender had no effect on functional activity in our study. Bergamini et al. found that older Italian men and women showed similar prevalence of functional limitation, 31% and 28%, respectively [37]. However, Hairi et al. found that female gender was significantly associated with functional limitation; they suggested that disadvantages resulting from limited education may contribute to the greater physical disability and functional limitation burden experienced by older women in their study [35].

 Increased incidence of disability was associated with malnutrition in this study. These results are in agreement with Oliveira et al. [38] who assessed the relationship between nutritional status and indicators of functional capacity among recently hospitalized elderly in a general hospital, and showed that these indicators were significantly more deteriorated among the malnourished individuals [38]. Using mini nutritional assessment (MNA) and Barthel index on 123 resident elderly, Cereda et al. showed that the poorer functional status was associated with low nutrition [39]. Also, Chevalier et al., [40] in a study designed to estimate the prevalence of malnutrition in frail elders undergoing rehabilitation and the association between their nutritional status and physical function, showed that there was an interrelationship between nutritional and functional status. It has already been shown that malnutrition compromises the functional status of the individuals [40].

## 5. Conclusions

 Results in this study suggest that the hospitalized elderly are exposed to malnutrition, which emphasizes the importance of early identification of malnutrition among them. High nutritional risk was more with older age, rural areas, educated patients, and low income, while gender had no significant effect. Functional abilities were better with younger age but had no significant correlation with other sociodemographic variables. Malnourished hospitalized patients are candidates for functional impairment. Significant associations were noticed between both nutritional and functional status and specific sociodemographic variables. These interrelationships require further studies to elucidate. Also, it is necessary to pay special attention to functional capacity when planning nutritional care for this vulnerable group.

*Limitations of This Study. *The patients included in this study were a convenient sample from patients admitted to one hospital, so patients from distant areas were not included. The large percent of illiterate patients (84%) could have an impact on the study. Larger number of patients should be included in future studies.

## Figures and Tables

**Table 1:** Demographic data of patients.

	*N*	%
Age		
65 < 75	164	82
75 < 85	28	14
≥85	8	4
Gender		
Male	112	56
Female	88	44
Residence		
Rural	168	84
Urban	32	16

**Table 2:** Sociodemographic variables in studied patients.

Education		
Illiterate	168	84
Educated	32	16
Vocation		
Retired	64	32
Farmer	52	26
Housewife	72	36
Crafts man	12	6
Habits		
Exercise		
Yes	0	0
No	200	100
Sleep		
Satisfied	84	42
Not satisfied	116	58
Sexual activity		
No	156	78
Satisfied	8	4
Not satisfied	36	16
Substance abuse		
Yes	56	28
No	144	72
Marital status		
Yes	80	40
No	4	2
Widowed	112	56
Divorced	4	2
Children		
Yes	192	96
No	8	4
Presence of close friends		
Yes	136	68
No	64	32
Income		
Low	112	56
Moderate	88	44
High	0	0
Transportation		
Available	196	98
Not available	4	2

**Table 3:** Nutritional and functional assessment of studied patients.

Nutritional assessment (NCL)	Good nutrition	52 patients	26%
	Moderate risk of malnutrition	36 patients	18%
	High risk of malnutrition	112 patients	56%

Functional assessment (BSI)	Range	8–20	
	Mean ± SD	16 ± 4.1	

**Table 4:** Association between nutritional checklist (NCL) and Barthel self-care index (BSI) with age.

	NCL						BSI	
	Good nutrition		Moderate risk		High risk		Range	Mean ± SD
	No.	%	No.	%	No.	%		
Age								
Young old (*N* = 164)	48	29.3	36	22.0	80	48.8	8–20	16.5 ± 3.84
Old old (*N* = 28)	4	14.3	0	0.0	24	85.7	8–20	13.4 ± 5.2
Oldest old (*N* = 8)	0	0	0	0	8	100	15–18	16.5 ± 2.1

*X* ^2^	10.4						3.65	
*P*	0.001						0.018	

**Table 5:** Association between nutritional checklist (NCL) and Barthel self-care index (BSI) with residence.

	NCL						BSI	
	Good nutrition		Moderate risk		High risk		Range	Mean ± SD
	*N*	%	*N*	%	*N*	%		
Residence								
Rural *N* = 168	40	23.8	24	14.3	104	61.9	8–20	15.6 ± 4.6
Urban *N* = 32	12	37.5	12	37.5	8	25	9–20	7.8 ± 3.67

*X* ^2^	8.26						1.81	
*P*	0.016						0.07	

**Table 6:** Association between nutritional checklist (NCL) and Barthel self-care index (BSI) with education.

	NCL						BSI	
	Good nutrition		Moderate risk		High risk		Range	Mean ± SD
	*N*	%	*N*	%	*N*	%		
Education								
Illiterate *N* = 168	52	31	28	16.7	88	52.4	8–20	15.97 ± 3.89
Educated *N* = 32	0	0	8	25	24	75	8–20	16.6 ± 4.9

*X* ^2^	6.7						0.85	
*P*	0.035						0.56	

**Table 7:** Association between nutritional checklist (NCL) and Barthel self-care index (BSI) with gender.

	NCL						BSI	
	Good nutrition		Moderate risk		High risk		Range	Mean ± SD
	*N*	%	*N*	%	*N*	%		
Gender								
Male *N* = 112	24	21.4	16	14.3	72	64.3	8–20	15.4 ± 4.4
Female *N* = 88	28	31.8	20	22.7	40	45.5	9–20	16.9 ± 3.4

*X* ^2^	3.56						4.35	
*P*	0.16						0.22	

**Table 8:** Association between nutritional checklist (NCL) and Barthel self-care index (BSI) with financial security (income).

	NCL						BSI	
	Good nutrition		Moderate risk		High risk		Range	Mean ± SD
	*N*	%	*N*	%	*N*	%		
Financial security (income)								
Low *N* = 112	20	17.9	24	21.4	68	60.7	8–20	15.8 ± 3.9
Moderate *N* = 88	36	40.9	8	9.1	44	50	8–20	16.4 ± 4.2

*X* ^2^	11.97						0.71	
*P*	0.017						0.47	

**Table 9:** Association between Barthel self-care index (BSI) with nutritional checklist (NCL).

	NCL			*F*	*P*
	Good nutrition	Moderate risk	High risk		
BSI					
*X* ± SD	18.4 ± 1.86	18 ± 2.4	14.5 ± 4.3	13.4	<0.001
Range	15–20	13–20	8–20		
